# State funding for cannabis research: an analysis of funding mechanisms and levels

**DOI:** 10.1186/s42238-025-00264-0

**Published:** 2025-03-14

**Authors:** Agnes Balla, Raymond G. Boyle, Christina Dempsey

**Affiliations:** 1 Research Policy Analysis and Coordination, University of California Office of the President, 1111 Franklin Street, 11th Floor, Oakland, CA 94607 USA; 2 Independent Health Policy Researcher, Minneapolis, MN USA; 3 Cannabis Policy Lab, Sacramento, CA USA

**Keywords:** Cannabis, Research, Funding, Adult use laws, Medical laws

## Abstract

**Background:**

This paper examines cannabis research funding across U.S. states that have legalized cannabis for medical or adult (non-medical) use. It specifically looks at state legislative efforts to fund cannabis research, and the amount and mechanisms used for funding distribution.

**Methods:**

We reviewed the text of legalization measures within states allowing medical or adult use (non-medical) cannabis for statutory language relating to research or scientific funding. When statutory language on research or scientific funding was not readily available or unclear in the legislative text, we reviewed state government websites or reference materials, or contacted state officials directly.

**Results:**

Overall, we found that 17 out of 38 states that have passed either medical or adult use laws have legislation that specify a funding mechanism for cannabis research. Of the 17 states that have legislation directing funding to research, only 12 have allocated funding to date. Of those states that have allocated funding, six states distributed funds directly to an academic institution and five moved funding first through state agencies. One state – California – distributed research funding to both an academic institution and through the state cannabis regulatory agency. The amount of funding varies significantly across the states.

**Conclusion:**

States have much to gain from scientific advancements in the cannabis field, especially as they navigate a preponderance of public policy issues without a federal structure to lean upon. However, with less than half of states that have legalized cannabis use in some form providing funding for research, there is a missed opportunity for states to increase understanding of the risks and benefits of cannabis use within their state. There is also a missed opportunity for researchers and cannabis regulators to collaborate on informing policy options and developing future evidence-informed cannabis regulations. There is a need for more states to consider adopting mechanisms to support cannabis research.

## Background

Cannabis has been classified as a Schedule I drug, the strictest categorization, since the enactment of the Controlled Substances Act in 1970. Despite the federal categorization, states have taken steps to implement laws allowing for medical and non-medical uses of cannabis as directed by ballot measures and, more recently, legislative action. Beginning in 1996, California became the first state to legalize cannabis with the passage of Proposition 215, which allowed patients with qualifying conditions to access cannabis as treatment. California’s Proposition 215 paved the way for more state cannabis laws, as seven additional states legalized medical cannabis use between 1998 and 2010.

The adoption of state cannabis use laws accelerated in later years, with pivotal measures in 2012 when Colorado and Washington became the first states to legalize adult (non-medical) use of cannabis. This marked the beginning of a shift away from seeing cannabis as a restricted drug. As of September 2024, cannabis is legal for medical use in 38 states, with 24 of those states also permitting adult (non-medical) use of cannabis. An additional 8 states have legalized low-THC/high-CBD forms of cannabis. In May 2024, the U.S. Department of Justice began rulemaking to reschedule cannabis from Schedule I to Schedule III.

Even though the legal landscape evolved, scientific understanding of cannabis and the impacts of legalization remains limited, hindering evidence-based public policy development. State cannabis regulators, tasked with implementing cannabis initiatives, have highlighted critical research gaps that hamper evidence-based decision making. For instance, state regulators have highlighted the need for research on cannabis consumption patterns, public health outcomes, and the effects of varying state regulatory frameworks (Schauer et al. 2023). Despite the need for research, it remains challenging to conduct research on cannabis and to fully understand the risks and benefits of legalization. Specifically, due to the federal classification of cannabis as a Schedule I substance, researchers must navigate obtaining approvals for conducting research from multiple federal agencies, use only federally approved sources for accessing cannabis for research use, and secure funding from a limited, often singularly-focused pool of sources. While the U.S. Department of Justice’s rulemaking to reschedule cannabis signals a potential reduction in barriers to conducting research, the need for research funding continues to persist.

Researchers have identified the lack of sufficient research funding as the top barrier in the cannabis field. In August 2022, the National Institutes of Health (NIH) released a Request for Information (RFI) seeking input from the scientific community on barriers to performing cannabis research (US DHHS 2022). In 2024, NIH reported that response data on the 2022 RFI showed increased funding opportunities as the highest need in the field (US DHHS (n.d.)).

To date, the federal government is the largest public funder of research in the United States (U.S.) and has provided most of the funding for U.S.-based cannabis research. The majority of these federal cannabis research dollars are distributed through the NIH. For example, NIH supported more than 785 cannabis research projects in 2021 with total grant funding of $369 million (US 2024). In comparison, during that same year, NIH provided $598 million for tobacco research and $568 million for alcohol research. The amount of federal funding allocated to cannabis research is considerably less than the funding provided for other research areas that have similar public health and public policy importance (US DHHS 2024). The NIH institute that funded the most cannabis research, by far, is the National Institute on Drug Abuse (NIDA) (Hellth.com 2020). NIDA-funded projects have traditionally focused on topics related to addiction, cannabis use, and its effects. More recently, NIDA prioritized funding for research that promotes approaches and best practices to develop and validate standard measures for evaluating cannabis and pain, substance use disorder, and comorbidities (US DHHS 2024).

Federal cannabis research funding has increased tenfold over the last two decades (Hellth.com. 2024) as government agencies at every level have grappled with difficult decisions about criminal law, product safety, and public health. However, the ability of researchers to generate timely, comprehensive data needed to inform policy changes have not kept pace with rapidly shifting cannabis policies and laws. This further underscores the need to close the funding gap through greater participation by state governments.

While federal funding of cannabis is known, less is known about how states fund cannabis research and the extent of opportunities that exist for state and university-level partnerships to strengthen opportunities to fund research on the scientific understanding of cannabis. This paper analyzes the current level of state government funding for cannabis research and the mechanisms used for funding distribution.

## Methods

### General overview

The National Conference of State Legislatures (NCSL) (National Conference of State Legislatures 2023) maintains an online comprehensive summary of all state medical marijuana and cannabis program laws, including links to relevant statutes and year of passage. This serves as a resource to search state regulated cannabis programs and current legalization status. Based on the NCSL database, we located the statutory text for medical or adult-use (non-medical) cannabis measures. Within these statutes, we specifically reviewed the language for provisions related to research funding, looking for key terms, including “research” and “scientific.” If the statutory text on research or scientific funding was not readily available or unclear, we supplemented our review by consulting state government websites or reference materials, or directly contacting state officials. In some cases, we reviewed legislative measures subsequent to legalization that were specific to state cannabis research efforts.

After reviewing statutory language relating to research or scientific funding, we built a database that included any funding specified in legislative text, the amount of funding, and a binary variable of whether the funding had been allocated to date. To determine the status of funding, we reviewed state government websites or reference materials, and if necessary asked state officials directly.

### Limitations of research

This analysis does not examine the content of cannabis legalization measures adopted by U.S. cities and counties, U.S. territories, Washington D.C., or jurisdictions outside of the U.S.

Further, this analysis focuses on funding specifically earmarked in legalization for the study of cannabis, rather than identifying all possible occasions where general state funds or agency budgets may be utilized in furtherance of cannabis research. Academic programs or institutions may receive generalized funding that is utilized, in whole or in part, to support its cannabis research projects. Similarly, state agencies, including cannabis agencies, may receive general budgetary support for staff that perform research-oriented work, such as data analysis and reporting, or may utilize general funding for relevant outside research. Neither of these ancillary, non-specific uses of funds were quantified or included in this research.

Finally, we gathered data from February 2024 to August 2024. Given the rapidly evolving cannabis legal landscape, some of the information we collected at the time may have been updated due to resolved lawsuits or more recently enacted legislation.

## Results

### General overview

As of September 2024, 38 states have legalized cannabis for either medical or adult use. Another eight states have legalized low THC/high CBD cannabinoid products. Only four states – Idaho, Kansas, Nebraska and Wisconsin – have not legalized medical cannabis, adult use cannabis, or low THC/high CBD cannabinoid products.

Of the 38 states that have legalized cannabis in some form (excluding low THC/high CBD states), 17 (45%) states have legislation specifying funding for research. Of the 14 states legalizing medical cannabis, 6 (43%) have adopted research funding mechanisms. Of the 24 states that have legalized both medical and adult use cannabis, 11 (46%) have adopted research funding mechanisms. No states that have legalized only low THC/high CBD formulations have created mechanisms to fund cannabis research. Table 1 provides an overview of these numbers.

**Table 1:** Snapshot of state cannabis legalization and adoption of research funding

Legalization Type	Number of States with Legalization	Number of States with Research Funding	Percentage of States with Research Funding
**Medical and Adult Use**	24	11	46%
**Medical Only**	14	6	43%
**Low THC/High CBD Only**	8	0	0%
**All Legalization Types (excluding Low THC/High CBD)**	38	17	45%

There are generally two ways in which states have allocated cannabis research funding: legislatively allocating funding directly to academic institutions or legislatively directing specific state agencies to fund research. Table 2 provides a comprehensive summary of each state’s legalization status, whether the state has legislation specifying research funding, whether the funding has been allocated, and what mechanism was specified in legislative text to allocate research funding.

**Table 2:** Comprehensive table of state cannabis legalization and adoption of research funding

State	Cannabis Legalization Status	Year and Method of Medical Legalization	Year and Method of Adult Use Legalization	Did legalization measure(s) establish a research funding mechanism?	Have funds been allocated?	What mechanism is used to fund research?
Alabama	Medical	2021 (Legislation)		Y	N	
Alaska	Medical and Adult Use	1998 (Ballot)	2014 (Ballot)	N		
Arizona	Medical and Adult Use	2010 (Ballot)	2020 (Ballot)	Y	Y	Through state agency
Arkansas	Medical	2016 (Ballot)		N		
California	Medical and Adult Use	1996 (Ballot)	2016 (Ballot)	Y	Y	Directly to academic institution and through state agency
Colorado	Medical and Adult Use	2000 (Ballot)	2012 (Ballot)	Y	Y	Directly to academic institution
Connecticut	Medical and Adult Use	2012 (Legislation)	2021 (Legislation)	N	N	Supports research in non-monetary ways
Delaware	Medical and Adult Use	2011 (Legislation)	2023 (Legislation)	N		
Florida	Medical	2016 (Ballot)		Y	Y	Directly to academic institution
Georgia	Low THC/CBD only			N		
Hawaii	Medical	2000 (Legislation)		N		
Idaho	None			N		
Illinois	Medical and Adult Use	2013 (Legislation)	2019 (Legislation)	Y	Y	Through a state agency
Indiana	Low THC/CBD only			N		
Iowa	Low THC/CBD only			N		
Kansas	None			N		
Kentucky	Medical	2023 (Legislation)		Y	Y	Directly to academic institution
Louisiana	Medical	2015 (Legislation)		N		
Maine	Medical and Adult Use	1999 (Ballot)	2016 (Ballot)	Y	N	
Maryland	Medical and Adult Use	2014 (Legislation)	2022 (Ballot)	N		
Massachusetts	Medical and Adult Use	2012 (Ballot)	2016 (Ballot)	N	N	Supports research in non-monetary ways
Michigan	Medical and Adult Use	2008 (Ballot)	2018 (Ballot)	Y	Y	Through a state agency
Minnesota	Medical and Adult Use	2014 (Legislation)	2023 (Legislation)	Y	Y	Directly to academic institution
Mississippi	Medical	2022 (Legislation)		N		
Missouri	Medical and Adult Use	2018 (Ballot)	2022 (Ballot)	N		
Montana	Medical and Adult Use	2004 (Ballot)	2020 (Ballot)	Y	Y	Through a state agency
Nebraska	None			N		
Nevada	Medical and Adult Use	1998 (Ballot)	2016 (Ballot)	N		
New Hampshire	Medical	2013 (Legislation)		N	N	Supports research in non-monetary ways
New Jersey	Medical and Adult Use	2010 (Legislation)	2020 (Ballot)	N		
New Mexico	Medical and Adult Use	2007 (Legislation)	2021 (Legislation)	N		
New York	Medical and Adult Use	2014 (Legislation)	2021 (Legislation)	Y	Y	Through state agency
North Carolina	Low THC/CBD only			N		
North Dakota	Medical	2016 (Ballot)		N		
Ohio	Medical and Adult Use	2016 (Legislation)	2023 (Ballot)	N		
Oklahoma	Medical	2018 (Ballot)		N		
Oregon	Medical and Adult Use	1998 (Ballot)	2014 (Ballot)	N		
Pennsylvania	Medical	2016 (Legislation)		Y	N	Supports research in non-monetary ways
Rhode Island	Medical and Adult Use	2006 (Legislation)	2022 (Legislation)	Y	N	
South Carolina	Low THC/CBD only			N		
South Dakota	Medical	2020 (Ballot)		N		
Tennessee	Low THC/CBD only			N		
Texas	Low THC/CBD only			N		
Utah	Medical	2018 (Ballot)		Y	Y	Directly to academic institution
Vermont	Medical and Adult Use	2004 (Legislation)	2020 (Legislation)	N		
Virginia	Medical and Adult Use	2020 (Legislation)	2021 (Legislation)	N		
Washington	Medical and Adult Use	1998 (Ballot)	2012 (Ballot)	Y	Y	Directly to academic institution
West Virginia	Medical	2017 (Legislation)		Y	N	
Wisconsin	None			N		
Wyoming	Low THC/CBD only			N		

### Examination of how states fund cannabis research

State cannabis research funding varies by status of allocations, distribution of funds, and overall program design. Though 17 states have adopted mechanisms to fund cannabis research, 5 have not allocated funding for the research intended. This leaves 12 states that have allocated research funding to date. Of those states that have allocated funding, six states distributed funds directly to an academic institution only and five states moved funding first through state agencies, which then provided those funds to researchers through grants and other programs. In addition, one state – California – distributed research funding in both ways. Overall, the amount of funding varies significantly across the states.

#### States that have allocated funding

States that have provided cannabis research funding have done so by either allocating funds directly to an academic institution or distributing cannabis research funds to researchers through a state agency, most commonly the state cannabis agency. This section describes the legislative mechanisms outlined by states that have allocated cannabis research funding.

##### Funding directly to academic institutions

In 2019, Florida passed Senate Bill (SB) 182 to provide $1.5 million to the Consortium for Medical Marijuana Clinical Outcomes Research (Florida 2019). SB 182 specified that the Consortium be housed in a state university and that the program produce information on the effects of medical marijuana use and the treatment of debilitating medical conditions with marijuana.

In 2022, Kentucky passed House Bill (HB) 604 to provide $2 million to the University of Kentucky to establish the Kentucky Center for Cannabis to conduct and fund research on the risks and benefits of cannabis use for certain medical conditions (Kentucky 2022).

In 2023, when Minnesota passed its adult use legislation, lawmakers set aside $2.5 million in annual funding from cannabis sales taxes to fund a center within the University of Minnesota School of Public Health (Kraker 2023). Minnesota lawmakers provided an additional $100,000 annually to the University of Minnesota for grants dedicated to cannabis genetics and agronomy research (Minnesota 2023). Also in 2023, Utah passed HB 230 to provide $650,000 for the University of Utah to establish the Center for Medical Cannabis Research to study the risks and benefits of cannabis use (Utah 2023).

Colorado and Washington focused their cannabis research funding efforts on furthering the work of their respective academic institutions. Since 2016, Colorado lawmakers have annually appropriated varying amounts of money from the Marijuana Tax Cash Fund to the Colorado State University System (CSU) to fund scientific and social science research at CSU-Pueblo concerning marijuana and other matters that impact the state (Colorado 2016). Washington’s 2012 adult-use ballot measure specified that six-tenths of 1% from the state’s cannabis excise taxes be directed to the University of Washington and four-tenths of 1% to Washington State University for research on the short and long-term effects of marijuana use, intoxication and impairment, and dissemination of such research (Washington 2011).

##### Funding through state agencies

Five states advanced research programs through funding provided to state agencies. Specifically, Arizona set forward legislative action to fund cannabis research through the state’s Department of Health Services’ (DHS) Arizona Biomedical Research Centre (ABRC). Arizona Revised Statute § 36–2822 requires that ABRC provide $5 million annually for five consecutive years from the medical marijuana fund for clinical trial grants (Arizona 2024). In addition, Arizona Revised Statute § 36–2812 requires that DHS provide funding, originating from a portion of marijuana excise tax, toward mental health research (Arizona 2023).

Illinois’s adult use legislation (HB1438) included language earmarking 2% of tax revenue for the Illinois Department of Human Services to carry out research-related activities (Illinois 2023). HB 1438 also created the Cannabis Business Development Fund, which can be used to fund research on the participation of minorities, women, veterans, or people with disabilities in the cannabis industry.

Michigan’s ballot measure to implement the state’s adult use law (Proposal 18 − 1) provided the Cannabis Regulatory Agency $20 million annually for a two-year period to fund research on the efficacy of marijuana in treating veterans and preventing veteran suicide (Michigan 2018). The Cannabis Regulatory Agency selected the University of Michigan and Wayne State University through Request for Proposal processes both years to carry out such research (Michigan 2024).

New York’s Marihuana Regulation and Taxation Act, which legalized adult use cannabis in the state in 2021, provides an annual disbursement of funds for reasonable costs incurred by the state to research and evaluate the effects of legalization (NY 2021). The research funded pursuant to the Act may be used to fund a range of topics, including public health impacts, public safety, and various economic impacts. To further promote research, the Office of Cannabis Management issued the Part 132 Research Regulations, which require researchers to obtain a license from the state when conducting research involving state regulated cannabis products that is not otherwise conducted pursuant to a federal Drug Enforcement Administration registration or federal Food and Drug Administration oversight (Cannabis Research License 2023). New York State has separately funded industrial hemp research by direct allocations to universities in the state as well as through the state’s Industrial Hemp Agricultural Research Pilot Program (New York 2020).

One state that specified legislative language for funding research through a state agency allocated funding unrelated to studying cannabis. Montana’s Initiative 190 provides monies from cannabis tax revenue to be used for certain programs, including research into nongame wildlife. The Montana Fish and Wildlife Commission recently approved a spending plan that includes 4% of marijuana sales tax revenues earmarked for nongame wildlife conservation (Larson 2023).

##### Funding to both academic institutions and through state agency

California provides research funding to an academic institution directly as well as through its state cannabis regulatory agency. In 2016, California voters approved the Adult Use of Marijuana Act (Proposition 64) to legalize cannabis adult use in the state. California’s Proposition 64 allocated $2 million annually to University of California San Diego’s Center for Medicinal Cannabis Research along with $10 million annually for a 10-year period to public universities in California as selected by the Department of Cannabis Control (DCC) through a Request for Proposal process. To date, DCC has allocated nearly $50 million to universities across the state for research projects on a range of topics relevant to public policy, including public health, criminal justice, public safety, economic impacts, environmental impact, and the effect of legalization on the cannabis industry (California 2024).

#### States that have not allocated funding

Five states – Alabama, Maine, Pennsylvania, Rhode Island, and West Virginia – adopted mechanisms to fund cannabis research but have not yet allocated funds. Alabama established a consortium for medical research, but lawsuits have stalled the work.

Maine’s Medical Marijuana Law was amended in 2018 to create a medical research grant program (Maine (2017)). However, to date, no rules have been promulgated and thus, no research or program funding has been established.

Chapter 19 of the Pennsylvania Medical Marijuana Act of 2016 established a research program to study the impact of medical marijuana available to those not participating in the state’s cannabis producer-academic partnership model so long as the research is approved by the federal Food and Drug Administration and federal Drug Enforcement Agency (Medical 2016). To date, Pennsylvania has not utilized the Chap. 19 provision.

Rhode Island established a marijuana trust fund intended to support cannabis research, but no monies from the fund have been utilized for research.

West Virginia’s legislation has a provision for the Office of Medical Cannabis to establish a research program, but a program has not been created to date.

#### Other support of cannabis research

Several states have supported cannabis research in non-monetary ways. For example, some states have adopted research agendas, commissioned or conducted policy research, or facilitated partnerships between state agencies and academic institutions.

Connecticut approved a program requiring researchers to apply to the state to conduct cannabis research, but the program does not currently provide funding for research.

Massachusetts made a concerted effort to fold in research as part of their state cannabis program. Specifically, Massachusetts required the Cannabis Control Commission to create a state research agenda to understand the social and economic trends of marijuana in the Commonwealth, inform future decisions aiding the closure of the illicit marketplace, and minimize the public health impacts of marijuana use. In a recent paper, Donahue and colleagues (Donahue et al. 2023) described the Massachusetts research agenda as a separate research department within part of the state Cannabis Control Commission.

New Hampshire created a commission of government officials to study and recommend a model that allows for full state control of sales and distribution of cannabis. The commission was administratively housed in the state Department of Justice, but without directed funding for the effort. The commission completed its report in December 2023.

While Pennsylvania has not directly funded research, it has created a cannabis producer-academic partnership model under Chap. 20 of the Pennsylvania Medical Marijuana Act of 2016 (Medical 2016). In this model, Academic Clinical Research Centers apply to the Pennsylvania Department of Health to collaborate with a medical cannabis producer, referred to as Clinical Registrants, to design research projects involving medical cannabis patients.

## Conclusion

The cannabis policy environment has rapidly evolved since 1996, when California first legalized medical cannabis use, as states have increasingly adopted decriminalization and legalization measures. Unfortunately, funding for cannabis research has not kept pace with the adoption of legalization. Federal government grants, the largest public source of research funding, have increased for cannabis in recent years but, as of 2021, was still 35% lower than for alcohol-related research and nearly 40% lower than for tobacco-related research.

Though cannabis products are legal in 38 states for medical use and 24 states for non-medical adult use, only 12 states have provided direct funding for cannabis research. This lack of investment is a missed opportunity for states to foster research about the impact of legalization and to increase understanding of the risks and benefits of cannabis use within their state. There is also a missed opportunity for collaborations between researchers and cannabis regulators to inform legislative discussion and develop future evidence-based cannabis laws.

Without federal participation or support, states are carrying the bulk of responsibility for regulatory oversight of cannabis. Given this environment, states have a unique opportunity to add to the scientific knowledge about cannabis, especially as they continue to form laws and regulations around cannabis products. Several states have been intentional in their efforts to incorporate research into policymaking efforts. For example, California and Colorado have identified specific policy knowledge needs over time and included such needs in recent funding opportunities for academic institutions. However, most states have not invested in research as a function of their cannabis legislation.

While there are a multitude of areas that need scientific attention, state cannabis regulators have published a proposed research agenda that, if implemented, would address critical research gaps that can advance informed and effective policies. Regulators identified six key areas for research support: medicinal cannabis use, product safety, consumer behaviors, policies to advance equity, youth prevention and public health and safety promotion, and policies to reduce illicit markets (Schauer et al. 2023).

There are many reasons states may not have previously chosen to invest in cannabis research. The current Schedule I designation under federal law is a significant challenge in the conduct of research, especially because the Schedule I classification prohibits researchers from working directly with cannabis sold in state sanctioned markets. This has likely had the effect of leaving states risk averse to research funding or unsure how to navigate complicated federal requirements imposed on research. In addition, the adoption of cannabis legalization is a complicated undertaking as states have to create and oversee a cannabis market. State regulators have to navigate legal medical and adult use, align with other product restrictions, such as alcohol and tobacco, while minimizing youth exposure to marketing and cannabis products.

States have much to gain from greater scientific understanding of cannabis and the impacts of legalization, and lawmakers should consider adopting mechanisms that would support research in parallel with legalization. This funding is important for investing in state infrastructure needed to support public agencies and academic institutions alike over time. The recent investments by Minnesota into their School of Public Health, and Utah’s provision of funds for medical cannabis research, are examples that should serve as encouragement for other states to adopt a cannabis research funding policy.

States that are interested in funding cannabis research could draw funds from cannabis-specific tax revenues, either in fixed annual amounts or at a specified percent of revenues collected. Fixed annual amounts would provide more consistency and predictability to academic institutions, which would better support institutional investments in necessary infrastructure. Percentages of tax revenues would result in more variability in research funding, with less money available when sales are lower but more money available if sales (and population cannabis use) increase. States can also support cannabis research in non-monetary ways, including by sharing regulatory market data with researchers and embedding researchers with cannabis agencies.

## Data Availability

The datasets created and analyzed during this study are available from the corresponding author upon reasonable request.

## Abbreviations

ABRC: Arizona Biomedical Research Centre
CBD: Cannabidiol
CSU: Colorado State University
DCC: Department of Cannabis Control
DHS: Department of Health Services
HB: House Bill
NIDA: National Institute on Drug Abuse
NIH: National Institutes of Health
RFI: Request for Information
SB: Senate Bill
THC: Tetrahydrocannabinol
U.S.: United States
